# Performance Comparison of Digital microRNA Profiling Technologies Applied on Human Breast Cancer Cell Lines

**DOI:** 10.1371/journal.pone.0075813

**Published:** 2013-10-08

**Authors:** Erik Knutsen, Tonje Fiskaa, Anita Ursvik, Tor Erik Jørgensen, Maria Perander, Eiliv Lund, Ole Morten Seternes, Steinar D. Johansen, Morten Andreassen

**Affiliations:** 1 Department of Medical Biology, Faculty of Health Sciences, University of Tromsø, Tromsø, Norway; 2 Department of Pharmacy, Faculty of Health Sciences, University of Tromsø, Tromsø, Norway; 3 Marine Genomics group, Faculty of Biosciences and Aquaculture, University of Nordland, Bodø, Norway; 4 Department of Community Medicine, Faculty of Health Sciences, University of Tromsø, Tromsø, Norway; Massachusetts General Hospital, United States of America

## Abstract

MicroRNA profiling represents an important first-step in deducting individual RNA-based regulatory function in a cell, tissue, or at a specific developmental stage. Currently there are several different platforms to choose from in order to make the initial miRNA profiles. In this study we investigate recently developed digital microRNA high-throughput technologies. Four different platforms were compared including next generation SOLiD ligation sequencing and Illumina HiSeq sequencing, hybridization-based NanoString nCounter, and miRCURY locked nucleic acid RT-qPCR. For all four technologies, full microRNA profiles were generated from human cell lines that represent noninvasive and invasive tumorigenic breast cancer. This study reports the correlation between platforms, as well as a more extensive analysis of the accuracy and sensitivity of data generated when using different platforms and important consideration when verifying results by the use of additional technologies. We found all the platforms to be highly capable for microRNA analysis. Furthermore, the two NGS platforms and RT-qPCR all have equally high sensitivity, and the fold change accuracy is independent of individual miRNA concentration for NGS and RT-qPCR. Based on these findings we propose new guidelines and considerations when performing microRNA profiling.

## Introduction

MicroRNAs (miRNAs) represent a class of small non-coding RNAs (ncRNAs), approximately 22 nucleotides (nt) in length, which regulate the expression of target genes at the posttranscriptional level [1]–[4]. MiRNAs contribute to important biological processes including cellular differentiation, proliferation, and apoptosis [5]–[9]. Most miRNAs regulate gene expression by guiding effector protein complexes (RISC) through binding to complementary sequences in the 3′ untranslated region (UTR) of mRNAs, followed by subsequent inhibition of translation or destabilization of the target mRNA sequence [10], [11]. Conserved miRNA targets sites have been predicted in as many as two thirds of all human mRNAs [12]. Furthermore, one specific miRNA may target different mRNAs and one specific mRNA may be regulated by multiple miRNAs [13]–[16].

Aberrant miRNA expression may have serious consequences for the cell, and miRNA species have been found to be involved in the initiation and progression of many human diseases, including cancer [17], [18]. This makes miRNAs interesting candidates as biomarkers in human cancer [19]–[21]. Indeed, miRNA profiling has been shown to be an important approach in the molecular characterization of tumor subtypes and disease progression [22], [23]. Consequently, such profiling strategies can provide important guidance in the choice of treatment strategy, which ultimately can increase cancer patient survival [9], [24].

MiRNA profiling involves the measurement of the relative amount of expressed miRNAs in a sample [25]. There are three major technological approaches that dominate the research field: MiRNA profiling based on hybridization (microarrays and nCounter) [26]–[28], next generation sequencing (NGS) [29], and amplification (reverse transcription quantitative real time-PCR, RT-qPCR) [30]. Platform comparison studies have been performed by comparing miRNA expression profiles obtained by RT-qPCR- and NGS analyses to the more cost-efficient high-throughput microarray analyses [31]–[33]. These studies conclude that RT-qPCR and NGS have better sensitivity and accuracy than hybridization-based microarray analyses. The performance of different NGS platforms has also been compared [34]–[37]. Here, the absolute values for individual miRNAs differ between the platforms, even though their relative abundances remain constant. Differences in the library preparation protocols appear to be the main reason for this discrepancy. The nCounter platform was recently included in a comparison study where microarray, Illumina NGS platform, and RT-qPCR were evaluated [38]. However, as in many other platform comparison studies [31], [37]–[38], RT-qPCR is only include for verification thereby not allowing the full potential of the method in correlation studies between platforms. This approach may cause significant bias in the data generated as reference genes are required for normalization of the results. In our study, we profile more than 700 miRNA species by RT-qPCR and use the complete data set for normalization.

To work out the most reliable strategy for studying miRNA expression patterns in biological samples, we compare the performance of different profiling technologies. Breast cancer cell lines were chosen as a model system since aberrant expression of several miRNA species in breast tumor tissue has previously been demonstrated, reflecting the heterogeneous nature of this disease [39]–[40]. Recent established profiling platforms within each technology group were included in the comparison: The nCounter platform and miRCURY locked nucleic acid (miRCURY) as representatives for the hybridization and RT-qPCR based technologies, respectively, and Illumina HiSeq and SOLiD4 as representatives for the NGS technology. Each platform was compared and evaluated regarding sensitivity, accuracy, and flexibility. Compared to previous reports, our study confer additional strengths to such analyses by (1) including a high number of miRNAs in all platforms that gives an un-biased robust sensitivity comparison, (2) using a unique combination of miRNA profiling platforms, including two NGS platforms in combination with other profiling technologies and the novel nCounter platform, (3) giving a deeper analysis of fold change agreement across platforms, deducting clear guidelines regarding technical accuracy of observed fold changes, and (4) for all analysis we have adjusted for the numbers of detected miRNAs in order to give a nonbiased comparison of platforms. We conclude that cross-platform comparison studies are important in order to better understand the nature of the results gained from novel technologies.

## Materials and Methods

### Cell Cultures

All cell lines were obtained from the American Type Culture Collection (ATCC) and cultured in a humidified atmosphere at 37°C with 5% CO_2_. Hs 578T cells were maintained in Dulbecco’s Modified Eagle Medium (DMEM) (Life Technologies, Inc) supplemented with 10% fetal bovine serum (FBS) (EuroClone, Italy), 2 mM L-glutamine, 0,01 mg/ml insulin, 100 U/ml penicillin, and 100 µg/ml streptomycin (all from Sigma). Hs 578Bst cells were maintained in Hybri-Care Medium supplemented with 10% FBS, 100 U/ml penicillin, 100 µg/ml streptomycin, 1.5 g/L sodium bicarbonate, and 30 ng/ml mouse epidermal growth factor (EGF). AU565 cells were maintained in DMEM, supplemented with 10% FBS, 100 U/ml penicillin, 100 µg/ml streptomycin, and L-glutamine. SK-BR-3 cells were maintained in McCoys 5A medium, supplemented with 10% FBS, antibiotics, and L-glutamine. Cells were propagated in vitro for 5–8 passages (8×10^6^ cells at 80% confluence) prior to total RNA isolation.

### Total RNA Isolation and miRNA Enrichment

Total RNA was isolated from 8×10^6^ cells using TRIzol (Life Technologies, Inc), with prolonged precipitation and centrifugation steps in order to preserve the small RNA fraction. Total RNA quantification and integrity assessment were performed using Quant-iT assay (Life Technologies, Inc) and Agilent 2100 Bioanalyzer (Agilent Technologies), respectively. All RNA used in this study had RNA integrity number (RIN value) above 9.5. The miRNA fraction was isolated from total RNA samples by flashPAGE™ Fractionator (Life Technologies, Inc) or PureLink™ miRNA Isolation Kit (Life Technologies, Inc) according to the manufacturer instructions.

### miRCURY LNA Analysis

Approximately 40 ng (per replicate) of total RNA were used for MiRNA expression quantification using the miRCURY LNA™ Universal RT miRNA PCR system and the miRNA Ready-to-Use PCR Panels V2M (Exiqon, Denmark) according to the manufacturer recommendations. Real time PCR (RT-PCR) amplification followed by melt curve analysis was carried out on the Applied Biosystems 7500 RT-PCR platform. Raw Cq values were calculated with the SDS plate utility software v2.1 (Life Technologies, Inc) with automatic baseline setting and manual ΔRn threshold of 500 for all assays. Amplification curves for every reaction were manually inspected to confirm log-phase amplification. Cq values were adjusted according to interplate calibrators. Cq values of 36 or higher were set as background (Not Detected), outliers were manually removed, and fold change analysis where all performed using Microsoft© Excel (Microsoft Corp, Redmond, WA).

### SOLiD4 Next Generation Sequencing Analysis

Approximately 100 ng of small RNA enriched samples were subjected to adaptor ligation and subsequently cDNA synthesis. The cDNAs were size selected based on expected size of miRNA and adaptors (60–80 nt) using Novex® pre-cast gels (Invitrogen). The purified cDNAs underwent 18 cycles of PCR using barcoded primers. The PCR products were purified using PureLink™ PCR Micro Kit (Invitrogen) and analyzed for size and concentration on Agilent 2100 Bioanalyzer using DNA 1000 or DNA HS chips. Equal molar amount of each barcoded sample were pooled together in one library, which subsequently were used in emulsion PCR to a total concentration of 0.5 pM. Approximately 650 million enriched beads were deposited on a full glass slide for SOLiD4 sequencing. The obtained raw color-space data were analysed in CLC Genomics Workbench (CLCbio, Aarhus Denmark). Adaptors were trimmed, sequences were grouped, counted, and annotated against mature miRNA sequence references. Successful annotation of a miRNA was stringent and did not include substitutions or length heteroplasmy. Hence, no isomiRs were collected. From 192,296,821 raw sequence reads 24,081,459 reads were annotated as mature miRNA species.

### Illumina HiSeq Next Generation Sequencing Analysis

Total RNA was shipped to Eurofins MWG operons facility in Ebersberg, Germany. Barcoded small RNA libraries were created from 1 ug total RNA according to Illuminas TruSeq small RNA Sample Preparation Guide. Barcoded pre-trimmed sequences were imported to CLC Genomic Workbench and followed the same workflow as for SOLiD sequencing. Here, from 92,961,27 raw sequence reads, 28,885,488 reads were annotated as mature miRNA species.

All raw sequences (SOLiD and Illumina) were submitted to the National Center for Biotechnology Information (NCBI) Short Read Archive, study SRP022047.

### NanoString nCounter Analysis

Total RNA (150 ng) was shipped to the NanoString Technologies facility in Seattle, USA for nCounter® Human miRNA Expression Assay analysis. RNA was incubated in the presence of miRNA specific capture and reporter probes, and non-hybridized probes were removed and the purified hybridized complexes were immobilized and aligned for data collection as previously reported [28]. All samples were analysed in triplicates. To account for minor differences in hybridization and purification efficiencies raw data was adjusted using a technical normalization factor calculated from six internal positive spike controls present in each reaction. Background hybridization was corrected by deducting the negative control mean plus two standard deviations calculated from eight negative controls.

### Data Normalization

A significant challenge when analyzing and comparing data is the difference in output generated by the various platforms. Therefore the relative expression of combinations of cell lines was used. Several normalization strategy methods, which including, global mean normalization [41], quantile normalization [42], linear total count scaling, and Trimmed Mean of M component normalization [43], were tested for the different data sets. We, however, found that implementation of different normalization strategies for the different platforms had a negative impact on the concordance between miRNA profiles (data not shown). In order to reduce normalization based bias we chose a single normalization strategy, linear total count scaling, for all four platforms. Since RT-qPCR operates with logarithmic numbers, normalization was achieved by linearization of the inverted expression value using the cut off value as the zero base-line. After normalization RT-qPCR expression values were converted back into log_2_ Cq values.

### Exact Mature miRNA Sequence Data Base (Exma-miRDB)

In order to compare the results obtained by the PCR, hybridization, and NGS technologies, we created an in house reference based on a modified version miRBase v17 that includes only mature human miRNA sequences. MiRNAs with identical mature sequence, but originated from different genomic loci, were merged. Similarly, miRNAs with different sequences but undistinguishable in one or more technologies, were also merged. The hybridization-based nCounter assay included 664 specific probes for human miRNAs. Some of these targets were in updated revisions of miRBase found to be obsolete and certain miRNA species were indistinguishable (e.g. hsa-mir-17 from hsa-mir-106a) in this technology. Consequently, 33 targets were either merged or excluded from this panel (Table S1). The prefabricated human miRNA panels for RT-qPCR contained originally 742 miRNA specific primers. Here, 20 miRNAs were obsolete and discarded. The updated screening panels for the four different platforms targets 631 (nCounter), 722 (miRCURY), and 1719 (SOLiD and Illumina) miRNAs (Table 1). Out of these, 517 were found to be concordant and hence preferred in the cross-platform comparison. However, in individual platform analyses that include technical correlation studies and side-by-side comparison, all mutual miRNA targets respective to the platforms in question were included.

**Table 1:** Platform screening potential.

	Targeted miRNAs	ObservedmiRNA	Percentage of reference
SOLiD	1719	748 (44%)	44%
Illumina	1719	630 (37%)	37%
nCounter	631	250 (40%)	15%
miRCURY	722	424 (59%)	24%

The theoretical number of possible miRNAs detected within each technology is compared to the observed number found in this study. IsomiRs are not included.

## Results

### Reference Datasets

**Figure 1:**
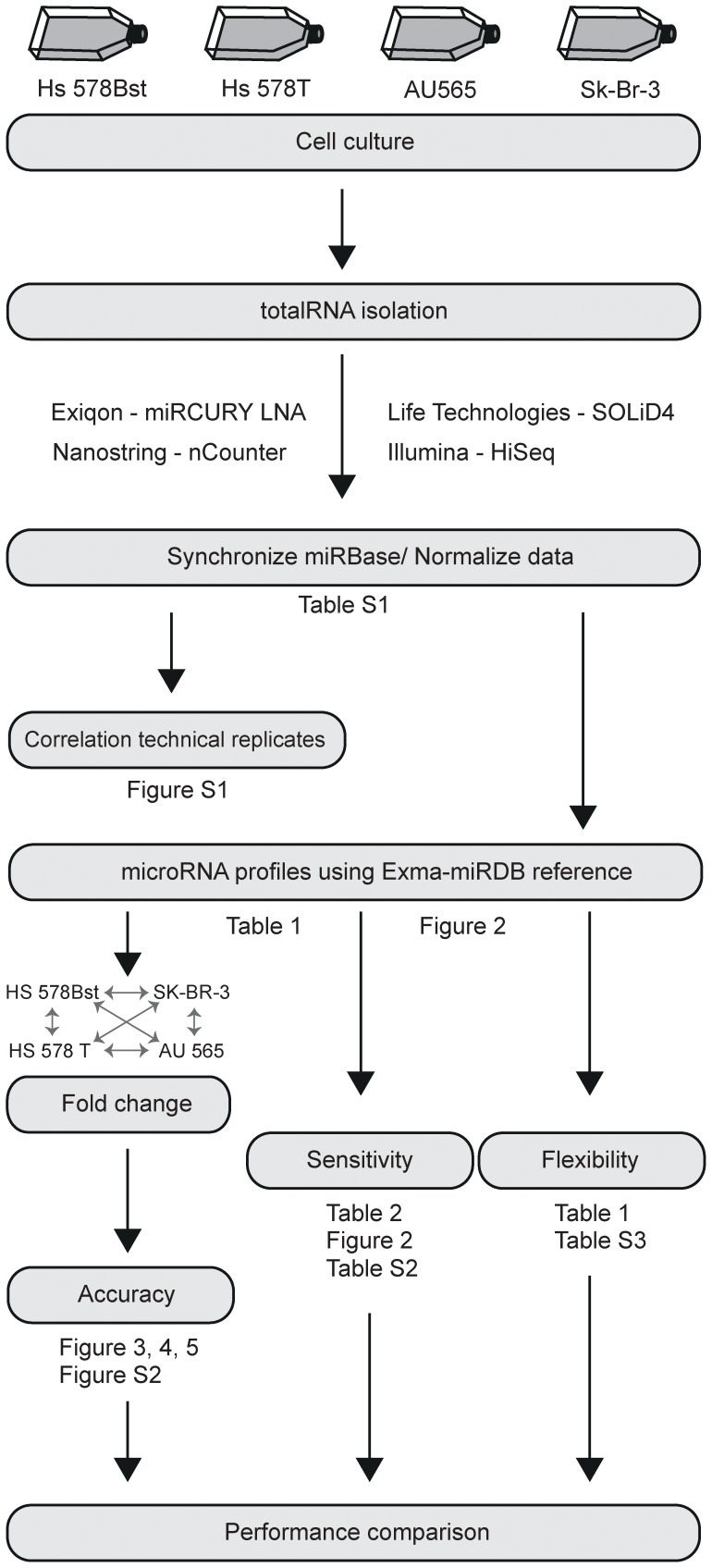
Project design. Relevant figure and table references are noted. Human breast cell lines were cultured and total RNA was extracted. MiRNA profiles were obtained using four different platforms; Exiqon miRCURY LNA, Life Technologies SOLiD4, Illumina HiSeq, and NanoString nCounter. A local miRNA database (Exma-miRDB) was generated based on mature sequences found in miRBase v17. The performances of the platforms were evaluated in regards to accuracy, sensitivity, and flexibility.

In this study three main technologies were used: (1) RT-qPCR (Exiqon miRCURY LNA), (2) RNA deep sequencing, NGS (Life Technologies SOLiD4 and Illumina HiSeq), and (3) hybridization (NanoString nCounter) (Figure 1). In order to compare the different output formats, a mutual reference database was generated. Exact mature miRNA sequences were extracted from the human miRNA database (miRBase v17, http://www.mirbase.org/) in order to create an in-house local database (Exma-miRDB). The final Exma-miRDB contained 1719 unique mature human miRNA sequences.

### MiRNA Data Generation

To evaluate the performance of the different technologies, miRNA expression profiling was carried out on four human breast cell lines; Hs 578Bst, Hs 578T, SK-BR-3, and AU565. Hs 578T is a triple-negative basal-like breast cancer cell line originally isolated from an infiltrating ductal carcinoma. Hs 578Bst was derived from the same patient, but isolated from normal breast tissue of an apparent myoepithelial origin. SK-BR-3 and AU565 were luminal-type breast cancer cell lines derived from a pleural effusion of a patient with breast carcinoma. Different cell lines derived from tumorgenic and healthy tissues in the same patient are of particular interest in miRNA profiling studies, as miRNA profiles are less biased towards genetic differences. MiRNA expression profiles were generated for all cell lines using the four different platforms described above and using the Exma-miRDB as reference.

Data generated from NGS covered the complete reference of 1719 miRNAs. Here, SOLiD sequencing detected 748 miRNAs in at least one cell line and 313 in all four cell lines. Corresponding values for Illumina HiSeq were 630 and 252, respectively. Of the 631 targeted miRNAs in the nCounter assay, 250 miRNAs were detected in one or more cell lines, while 113 miRNAs were found in all four cell lines. RT-qPCR quantification detected 424 (one or more cell line) and 173 miRNAs (all four) out of the 722 different targets assessed by this platform (Table 1).

Technical reproducibility analyses were carried out for three of the platforms (miRCURY, nCounter, and SOLiD) by analyzing linear relationships between all combinations of replicates within each cell line. Pearson’s correlation analysis revealed a very high level of reproducibility; R = 0.997±0.010 (miRCURY, three replicates), R = 0.992±0.008 (nCounter, three replicates), and R = 0.924±0.026 (SOLiD, two replicates) (Figure S1). These datasets, including data from Illumina, were used in the miRNA expression data comparison to evaluate the performance of the different platforms in terms of sensitivity, accuracy, and flexibility.

### Sensitivity

The sensitivity of a platform was defined as the ability to detect miRNAs present in a biological sample. The sensitivity is simply calculated by dividing the number of detected true positive miRNAs with the total number of true positive miRNAs in a sample. Since the investigated biological samples were isolates from cell lines, and not synthetic miRNA species, the true miRNA counts in our samples were unknown. Therefore, to calculate the sensitivity a miRNA was defined as a true positive if at least three out of the four platforms identified the miRNA, and as a true negative/absent if identified by only two or less platforms. MiRNA profiles generated from all cell lines were used, but only miRNAs screened for in all platforms (517) were included in the comparison. Using the 517 concordant miRNAs and the four cell lines gave rise to a total of 2068 miRNA data points in the sensitivity analysis. Out of these 777 miRNAs (38%) were regarded as true positive based on the above-mentioned criteria. Here, 763 and 764 were detected by the NGS platforms resulting in a sensitivity rating as high as 0.982 and 0.983, SOLiD and Illumina, respectively (Table S2). miRCURY (RT-qPCR) came close to the NGS platforms with a sensitivity of 0.959 (745 detected). nCounter (hybridization), on the contrary, detected only 501 of the miRNAs, which gave a sensitivity of 0.645.

**Figure 2:**
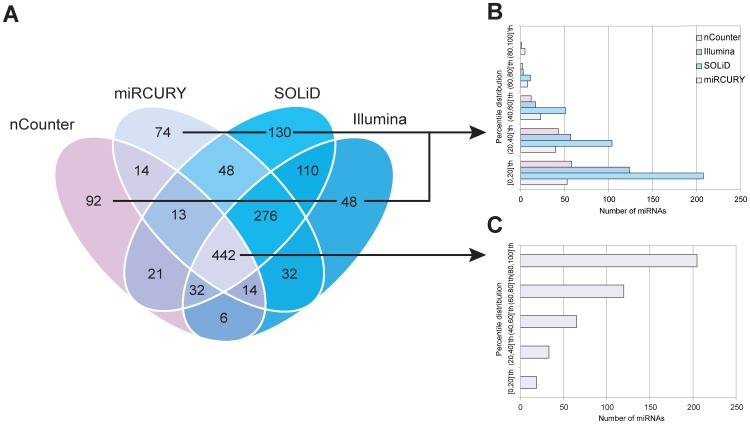
Platform sensitivity. (**A**) Venn diagram displaying the convergence of detected miRNAs by the four platforms. Dispersion of the concentration of individual miRNAs detected by a single platform (**B**) and all platforms (**C**). The miRNAs are grouped in accordance to the percentile distribution, where the 20% lowest expressed miRNAs within a platform are grouped, the miRNAs with an expression between the 20% lowest and the 40% lowest are grouped, and so on.

The degree of convergence between each platform was then examined (Figure 2A). We found 442 miRNAs to be detected by all four platforms in the four cell lines. Additionally, 910 miRNAs throughout the cell lines were detected by at least one single platform. The percentile distributions of the individual miRNAs within these two subsets were grouped according to their individual concentration. Not surprisingly, the expression percentile distribution clearly illustrated the correlation of individual miRNA sample concentration and the ability to be identified by the various platforms. The majority of miRNAs detected by only a single platform were expressed at low levels, whereas the majority of highly expressed miRNAs were detected by all four platforms (Figures 2B and 2C). This is consistent with a postulate that the probability to be detected by the various platforms is higher for an extensively expressed miRNA than from a scarcely expressed miRNA. In addition, the probability of an expressed miRNA to be a false positive decrease as other platforms detects the same miRNA. Together, these two postulations were used to create a new weighted sensitivity comparison. Here, a highly expressed miRNA detected by several of the platforms generated a higher score, (positive if it were detected by both platforms, and negative if it were only detected by one of the platforms), than a scarcely expressed miRNA detected by few platforms. To overcome the problem of having to create a hypothetical list of true and false miRNAs in our samples, two and two platforms were grouped in order to evaluate the sensitivity in pairs. The calculation was done accordingly: 1) All miRNA were used to assess if the pair under investigation detected the miRNA or not. If both members identified the same miRNA it was given a positive value. In contrast, if only one member detected the miRNA, it was given a negative value. 2) The score was weighted in accordance to the expression of the miRNA and in accordance to detection of the miRNA by the two other platforms not under investigation. 3) The total score was scaled in order to compare the different pair combinations; a score of −1.000 corresponds to no commonly detected miRNAs, while a score of 1.000 were given if both team members detected the exact same set of miRNAs.

The result shows that every time nCounter is teamed up with another platform, the sensitivity falls below the average of all sensitivity comparisons. In contrast, all of the other platforms perform very well in other pair conformation; the best being the two NGS platforms (SOLiD and Illumina), followed by any combinations of NGS and miRCURY (Table 2).

**Table 2:** Platform sensitivity.

	nCounter paired miRCURY	nCounterpaired SOLiD	nCounterpaired Illumina	miRCURY paired SOLiD	miRCURY paired Illumina	SOLiD paired Illumina
**miRNA detected in a single platform**	**581**	**690**	**606**	**427**	**345**	**312**
…no additional platforms (−)	166	222	140	204	122	178
…one additional platform (−−)	107	178	177	177	178	107
…two additional platforms (−−−)	308	290	289	46	45	27
**miRNA detected in both** **platforms**	**483**	**508**	**494**	**779**	**764**	**860**
…only these two platforms (+)	14	21	6	48	32	110
…one additional platform (++)	27	45	46	289	290	308
…two additional platforms (+++)	442	442	442	442	442	442
**% miRNA detected by both platforms**	**45%**	**42%**	**45%**	**65%**	**69%**	**73%**
**Weighted score**	**0.31**	**0.30**	**0.31**	**0.74**	**0.75**	**0.83**

For each combination of platforms the identified miRNAs were used to calculate a weighted detection score based on the concordance of the two platforms (noted by “+” and “−”). The score took into account both the expression value of the individual miRNAs and detection by the other platforms that were not under investigation. The weighted score range from −1.0 (no agreement) to 1.0 (full agreement). Only miRNAs screened for in all platforms were included.

### Accuracy

The accuracy of a platform was defined as the ability of the platform to correctly identify fold change differences in biological samples. In order to evaluate the accuracy, the relative expression levels of miRNAs using all six combinations of the four cell lines were included. For assessment of the accuracy, pairs of platforms were compared. We found that for most miRNAs (average of 83%) the pattern of expression (up- or down-regulated) was similar, but the relative amplitude of the fold changes varied according to the different platforms being used.

**Figure 3:**
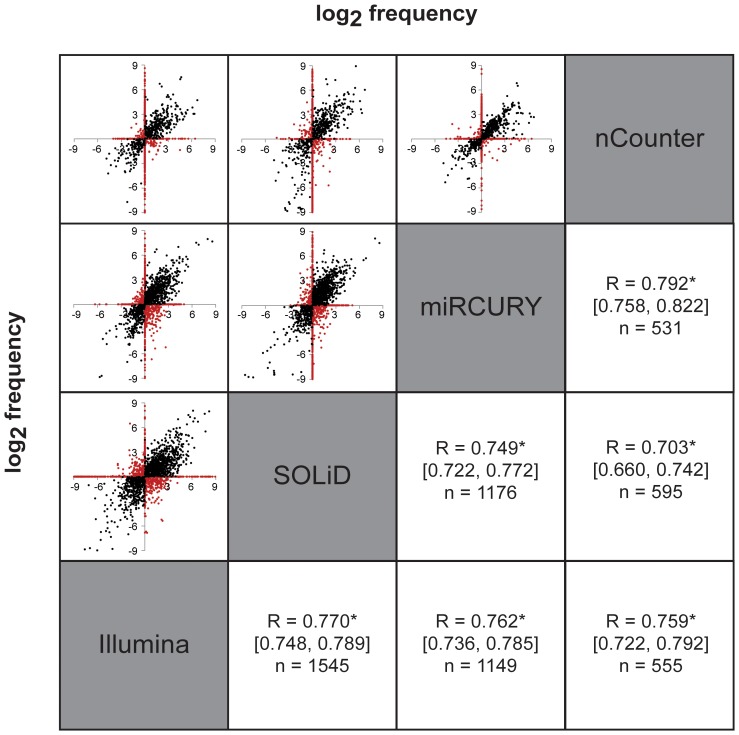
Fold change scatterplot. The miRNA fold change values are plotted for every combination of platforms. Fold change values were log_2_ transformed and Pearson’s correlation (R) was used to assess the accuracy. Confidence limits are included in brackets. Number of miRNA included in the calculation (n). Asterisk (*) indicate p-value <0,0001.

Pearson’s correlation (R) was used to calculate the accuracy across platforms. R-values were in accordance to the calculation on the pattern of expression, and showed that the overall correlation for all platforms was high (P = 0.703–0.797) (Figure 3). However, we were not able to identify a specific pair of platforms as significantly better than any of the other combinations of platforms. These results are in accordance with previously published platform comparison performances [31]–[32], [44]–[46]. We further noted that the number of data points included in the correlation differed significantly between the technologies, from 516 (miRCURY/nCounter) to 1545 (SOLiD/Illumina). These differences were not only due to the limited number of primers and probes in the miRCURY or nCounter panels, but were also a result of the ability of NGS to identify more miRNAs in general, as seen by the sensitivity comparison (Table 2, Figure S2).

**Figure 4:**
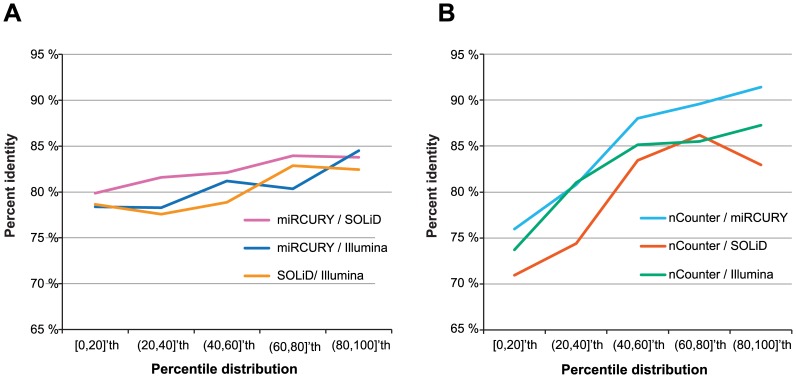
Platform accuracy in relation to miRNA concentration. (**A**) The percent identity in fold change across the percentile distribution of miRNAs for all platform combinations without the nCounter platform. Here, an even accuracy is seen across the full range of miRNA concentration. (**B**) The same data for platform combinations involving the nCounter platform reveal a large drop in accuracy when the miRNA abundance is low.

To further examine the accuracy, the fold change in relation to miRNA expression was investigated. The accuracy was found to be constant across the concentration of individual miRNAs for the NGS platforms and for miRCURY (Figure 4A **)**, with an average of 81% ±2%. A higher variation was seen for the comparisons including the nCounter platform (82% ±6%), with the accuracy being proportional with increasing miRNA sample concentration (Figure 4B).

**Figure 5:**
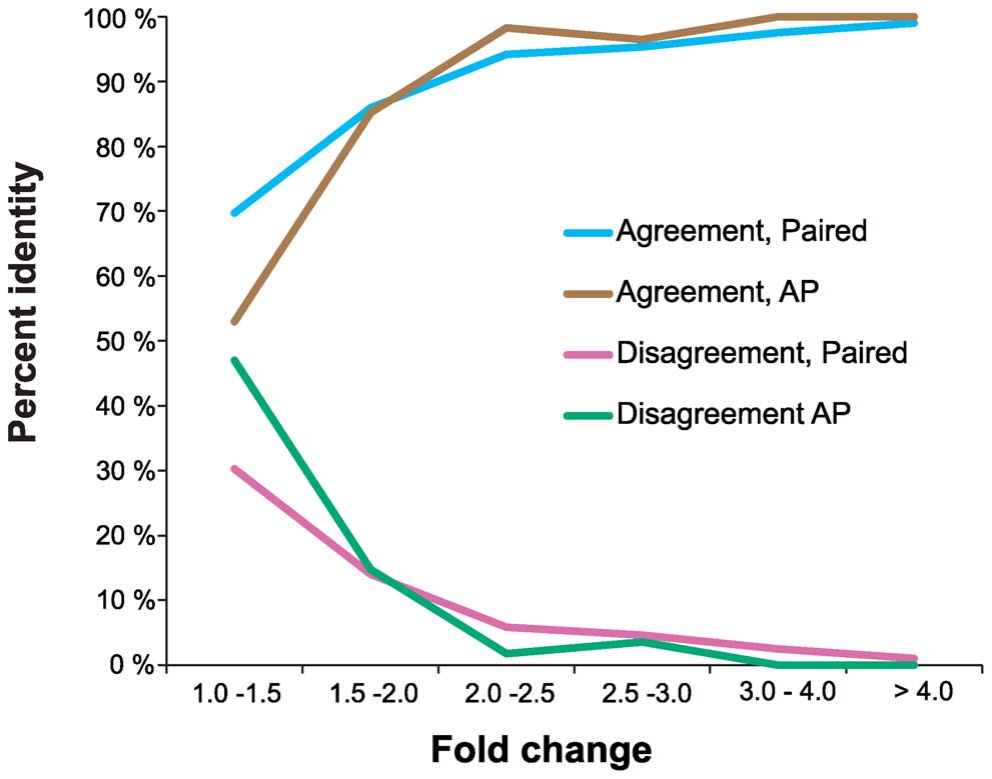
Platform accuracy across fold change level. Percent identity across the fold change level for every combination of platforms (Paired) and for miRNAs only mutually detected by all platforms (AP).

Platform dependent differences were not identified when analyzing the data in accordance to fold change value, but a general trend was seen for all platforms. If the change was three fold or more it was only a 2% likelihood of the fold change to be contradictable when comparing the platforms in pairs (Figure 5, Paired). However, if the fold change was close to one, 30% of fold changes were found to be contradictory (Figure 5, Paired). When exclusively examining the fold changes for miRNAs that were mutually detected by all four platforms (AP) (Figure 5, AP, Figure S2), nearly half (47%) of the miRNAs with an average fold change close to one had at least one platform showing a contradictory fold change. In contrast, all platforms were in agreement if changes were above three fold. Thus, we conclude that the probability of correctly identifying a true difference in expression increases with the level of fold change.

### Flexibility

The flexibility of a platform reflects its ability to serve additional functions to the data collected. In this context, there are several reasons for why we find NGS technology far more versatile than RT-qPCR and hybridization based technologies. (i) In profiling, NGS has the advantage of being the only technology that uses the complete reference dataset. (ii) NGS is collecting data of miRNA species not yet discovered or annotated in the reference data base. RT-qPCR and hybridization technologies, however, are restricted to pre-designed primers and probes combinations, and (iii) due to the single nucleotide resolution of the NGS platforms, additional level of information is collected in both length and site heteroplasmy for each miRNA (isomiRs). By a simple search in our SOLiD and Illumina datasets, we found approximately the same number of isomiRs reads as mature miRNAs reads (Table S3). We also find that sequences annotated as isomiRs behaved in a similar manner as sequences annotated as canonical mature sequences in regards to accuracy (Figure S3).

## Discussion

### Performance Evaluation

The strength and limitations for the main miRNA profiling technologies, as well as for the individual commercial vendors, have been thoroughly reviewed in [25]. In this work, however, we challenge their conclusion regarding that RT-qPCR has a better sensitivity than NGS. The two NGS platforms were here found to have the highest sensitivity score. This was due to the fact that they detected the highest number of miRNAs, which are likely to be true positives as these miRNAs were also found to be expressed by additional platforms. miRCURY also performed very well, and when combined with the NGS platforms no obvious preference for either SOLiD or Illumina could be observed. Surprisingly, in contrast to NGS and miRCURY, the nCounter system was found to have a low sensitivity. nCounter is a hybridization based technology and the only platform in our study lacking an amplification step. This may reduce the window between a true-positive miRNA expression and the background, which thereby accounts for at least some of the reduction in the sensitivity. This was clearly visualized in the Venn diagram presented in Figure 2. Here, miRCURY and the two NGS platforms detect 276 miRNAs, which were not identified by nCounter, a number that is about half of the miRNAs detected by all four platforms (442 miRNAs). Hybridization based technologies, in general, have limitations in distinguishing between highly similar target sequences [47]. Indeed nCounter has this limitation as well, particularly if the dissimilarity is located at the 5′ end of the miRNA. As for miRNA profiling there are lots of miRNA species with only one nucleotide difference, including the highly cancer relevant hsa-miR-17 and hsa-miR-106 (see Table S1). However, in projects that involve large sample size and limited number of highly expressed non-merged miRNAs, we find the nCounter system to be highly capable due to its short hands-on time [25].

The accuracy was found to be similar for all combinations of the four platforms. A slightly, but not significantly, better correlation was seen for the nCounter system in combination with miRCURY and for the combination of the two NGS platforms SOLiD and Illumina. This could be due to a more similar library preparation protocol between these platforms, which would be in accordance to previous reports that library preparation method, and not the sequencing platforms, appears crucial in miRNA expression profiles [34], [36]–[37]. One of these studies also concluded that different library preparation methods gave different expression ranks for the miRNAs detected [34]. Thus, the absolute expression level cannot exactly be determent for any of the platforms. The difference in miRNA rank outcome from library preparation will not however affect the fold change as the same bias is introduced for both the control and test sample. Here we show that the accuracy of NGS and miRCURY is close to constant across the individual miRNA concentrations (Figure 4). This means that in a fold change comparisons there is not necessary to discard scarcely expressed miRNAs involved in large fold changes as it is the change itself and not the individual expressions that are comparable across platforms. However, the expression levels will affect the probability for detecting the miRNA by a different platform. This is highly important in verification studies where miRNAs that do not have large fold changes or are highly expressed may fail verification.

When is a miRNA gene differentially expressed? A common practice in profiling studies is to score a miRNA as differentially expressed if the miRNA level shows change above two fold. This threshold might be sufficient to hide biological significant differences. However, due to technical limitations of profiling technologies and due to normal biological variations, this threshold might in fact also be too low in order to avoid false positive. We tested for normal variation by performing an additional independent experiment that included a biological replicate of the Hs 578T cell line. Here, we detected a median fluctuation of 2.6 fold change between identical miRNA when compared to our original sequencing experiment (Table S4). These data are supported by the observation of 2–4 fold random fluctuations for many genes in yeast [48], [49]. In our study, we see a technical agreement of only 81% at a fold change level below two fold. We therefore conclude based on the combination of both technical and biological variations that required level of fold change should be increased to a change of at least 3–4 fold for a miRNA to be defined as differentially expressed.

Only NGS platforms are able to detect isomiRs. Based on our data analysis of the four cell lines, the abundance of isomiRs is about equal to the amount of mature sequences (Table S3). As more NGS miRNA profiling studies are being performed, the mature sequences of present known miRNAs will probably be redefined when isomiRs are discovered to be more dominantly expressed than the canonical miRNA. The role of isomiRs has still not been unraveled, but an increasingly number of recent reports suggests important new and distinct functions for the isomeric miRNAs compared to their canonical counterparts [50], [51]. In this study we build up on the statement of isomiR being real miRNA variants and not sequencing errors by showing a similar behavior for isomiRs and conical miRNAs in regards to profiling accuracy for SOLiD and Illumina.

## Concluding Remarks

Based on the sensitivity and accuracy obtained in this study of the different platforms, we recommend an initial miRNA profiling based on NGS or RT-qPCR. Furthermore, NGS has additional strengths in regards to the flexibility, and the SOLiD and Illumina platforms perform equally well. Recent developments in NGS technologies have lowered the cost and hands-on time of high-throughput profiling to a level comparable to RT-qPCR. These considerations give in our view NGS an important advantage in miRNA profiling. Regardless of the technology or platform used, we strongly recommend that biological relevant miRNA should be verified by an independent platform, and that expression differences should be supported by a high fold change.

## Supporting Information

Figure S1 **Technical replicate scatter plots.** The combinations of every technical replicates were used to create the scatterplot. Correlation coefficients were calculated using Pearson’s correlation (R). MiRNA replicates with a fold change difference >2 are colored red. (**A**) miRCURY, three replicates (4263 data points), (**B**) nCounter, three replicates (2491 data points), (**C**) SOLiD, two replicates (1876 data points).
(TIF)

Figure S2 **Heat map of miRNAs detected in all four platforms.** Only the relative expressions found in all platforms from the combination of the cell line Hs 578Bst versus Hs 578T are shown. Fold change values are log_2_ transformed, and miRNAs are clustered according to hclust function (R-package). (**A**) Histogram showing the fold change distribution. (**B**) Green color represent a downregulation in Hs 578T compared to Hs 578Bst, and red color represent an upregulation in Hs 578T compared to Hs 578Bst. Hierarchical clustering was performed to display the data. Differentially expressed miRNAs that were reported by all platforms (>3 fold) are marked in bold.
(TIF)

Figure S3 **Next generation sequencing platform accuracy for canonical miRNAs versus isomiR.** The miRNA fold change values are plotted for the combination of SOLiD and Illumina for (**A**) canonical miRNAs and (**B**) isomiRs. Fold change values were log_2_ transformed and Pearson’s correlation (R) was used to assess the accuracy. Confidence limits are included in brackets. Number of miRNA included in the calculation (n). Asterisk (*) indicate p-value <0,0001. Platform accuracy in relation to miRNA concentration for the combination of SOLiD and Illumina for (**C**) canonical miRNAs and (**D**) isomiRs. The percent identity in fold change is plotted across the percentile distribution of miRNAs. Platform accuracy across fold change level for the combination of SOLiD and Illumina for (**E**) canonical miRNAs and (**F**) isomiRs.
(TIF)

Table S1 miRCURY and nCounter panlels updated according to miRBase v17. Changes are synchronized with the local miRNA database (Exma-miRDB).
(PDF)

Table S2 Sensitivity and specificity calculation. A positive miRNA was defined as a miRNA that were detected by at least 3 platforms. In total 777 miRNAs were defined as true positive and 1291 miRNAs were defined as true negatives. Only miRNAs screened for in all platforms were included. (A) nCounter, (B), miRCURY, (C) SOLiD, (D) Illumina, (E) Sensitivity and Specificity calculation.
(PDF)

Table S3 IsomiR quantitated in NGS data. NGS data were mapped against Exma-miRDB (includes only the mature miRNA sequences in miRBase v17), as well as the hairpin sequences of all annotated miRNA in miRBase v17. Both sets of NGS data generated from Illumina and SOLiD were used for isomiR quantification.
(PDF)

Table S4 MicroRNA profile of two biological replicates using SOLiD sequencing. Values are log2 transformed and the differences are presented as absolute fold change values.
(PDF)
