# Supplementary figures and images for: Performance Comparison of Digital microRNA Profiling Technologies Applied on Human Breast Cancer Cell Lines
Source: PLoS One. 2013 Oct 8;8(10):e75813. doi: 10.1371/journal.pone.0075813 (PMC3793004; doi:10.1371/journal.pone.0075813)

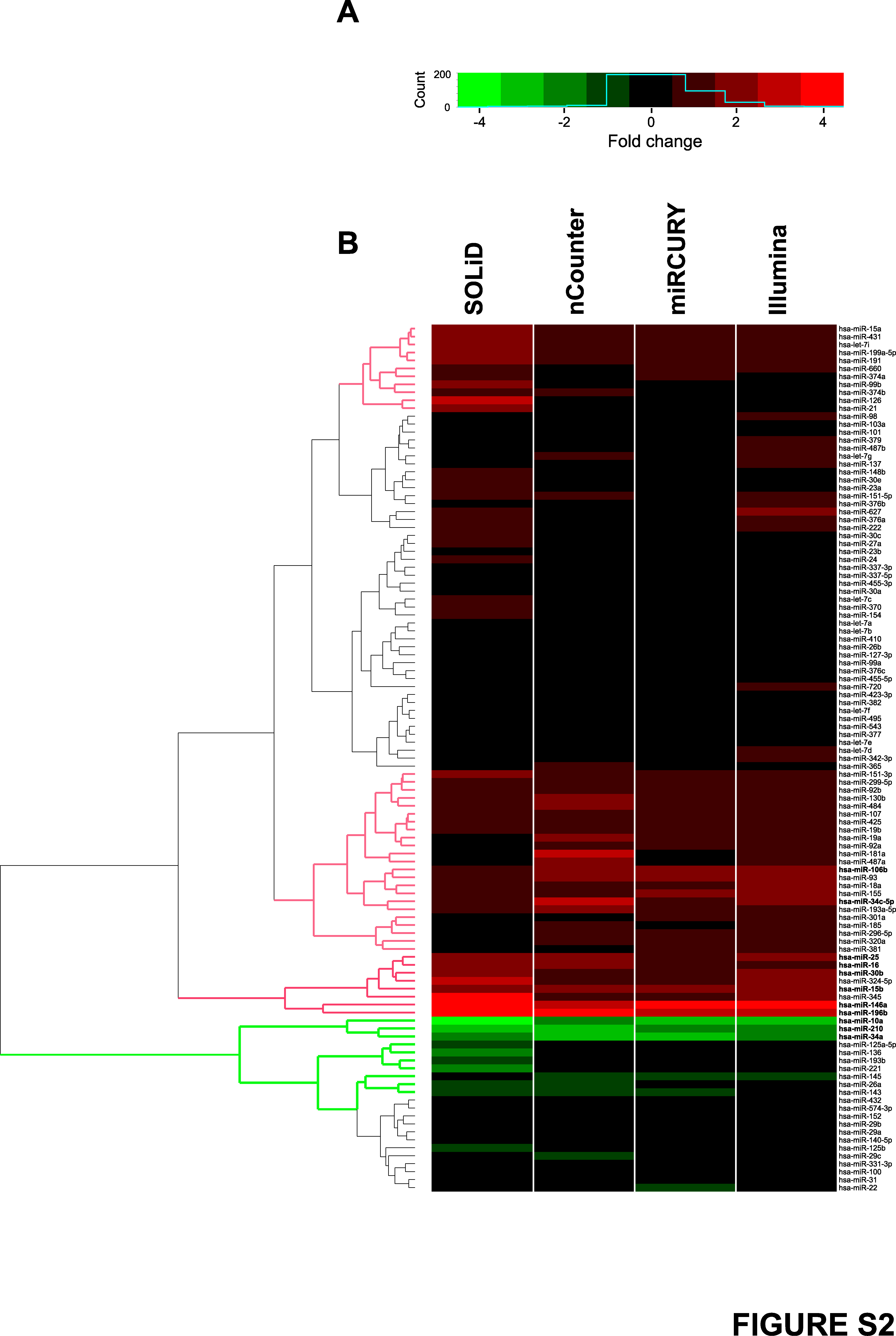

Supplement: Figure S2 — Heat map of miRNAs detected in all four platforms. Only the relative expressions found in all platforms from the combination of the cell line Hs 578Bst versus Hs 578T are shown. Fold change values are log2 transformed, and miRNAs are clustered according to hclust function (R-package). (A) Histogram showing the fold change distribution. (B) Green color represent a downregulation in Hs 578T compared to Hs 578Bst, and red color represent an upregulation in Hs 578T compared to Hs 578Bst. Hierarchical clustering was performed to display the data. Differentially expressed miRNAs that were reported by all platforms (>3 fold) are marked in bold. (TIF) [file pone.0075813.s002.tif]

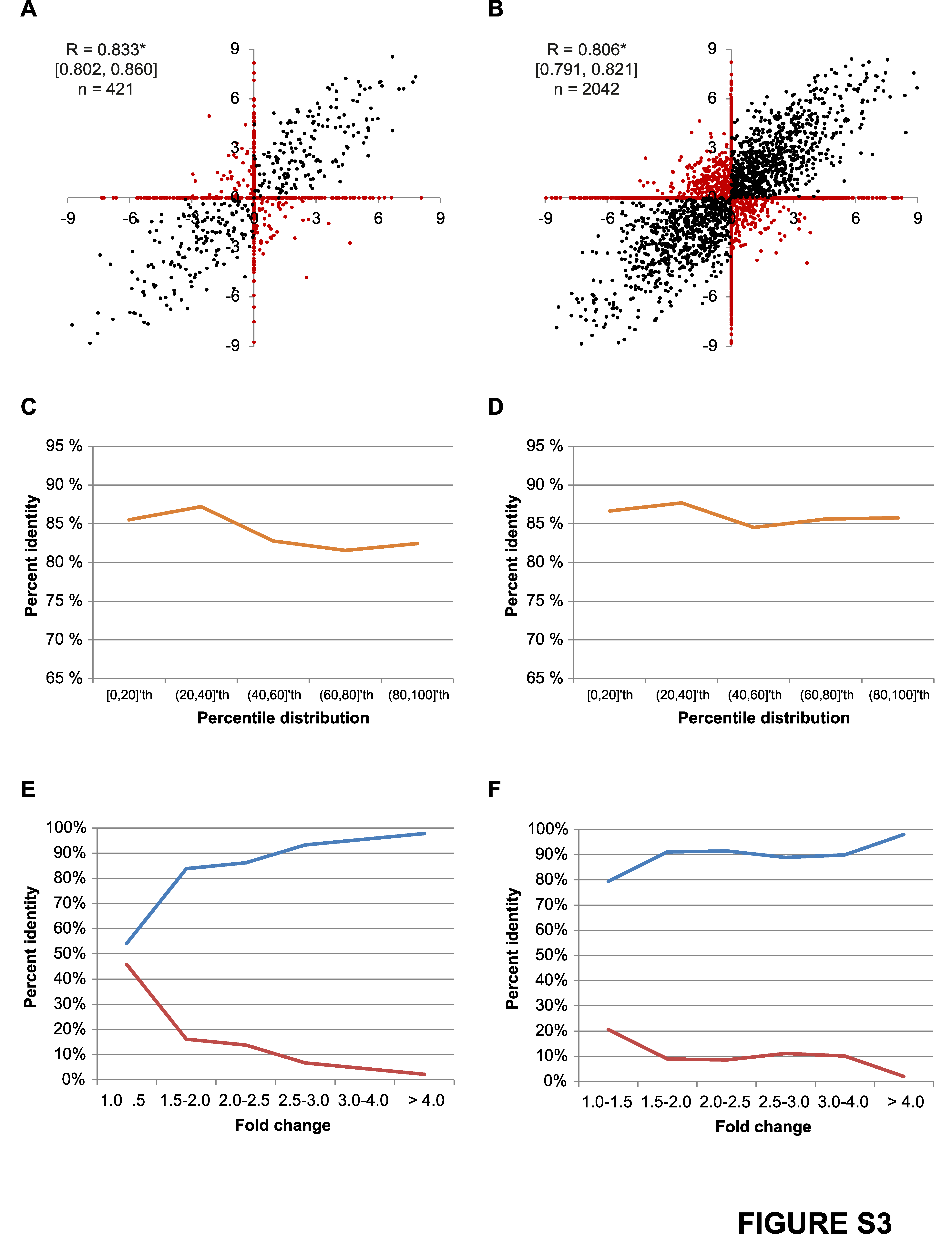

Supplement: Figure S3 — Next generation sequencing platform accuracy for canonical miRNAs versus isomiR. The miRNA fold change values are plotted for the combination of SOLiD and Illumina for (A) canonical miRNAs and (B) isomiRs. Fold change values were log2 transformed and Pearson’s correlation (R) was used to assess the accuracy. Confidence limits are included in brackets. Number of miRNA included in the calculation (n). Asterisk (*) indicate p-value <0,0001. Platform accuracy in relation to miRNA concentration for the combination of SOLiD and Illumina for (C) canonical miRNAs and (D) isomiRs. The percent identity in fold change is plotted across the percentile distribution of miRNAs. Platform accuracy across fold change level for the combination of SOLiD and Illumina for (E) canonical miRNAs and (F) isomiRs. (TIF) [file pone.0075813.s003.tif]
