# Supplementary material for: Performance Comparison of Digital microRNA Profiling Technologies Applied on Human Breast Cancer Cell Lines
Source: PLoS One. 2013 Oct 8;8(10):e75813. doi: 10.1371/journal.pone.0075813 (PMC3793004; doi:10.1371/journal.pone.0075813)
Supplement: Table S1 — miRCURY and nCounter panlels updated according to miRBase v17. Changes are synchronized with the local miRNA database (Exma-miRDB). (PDF) [file pone.0075813.s004.pdf]

Supplementary Table S1. miRCURY and nCounter panlels updated according to miRBase v17**.**
Changes are synchronized with the local miRNA database (Exma-miRDB).

miRCURY	nCounter	SOLiD / Illumina	Comment
hsa-let-7c_star			Deleted in mirbase 17
hsa-miR-103	hsa-miR-103	hsa-miR-103a	Renamed hsa-miR-103a
hsa-miR-103-2_star		hsa-miR-103a_star	Renamed hsa-miR-103a_star
hsa-miR-103-as		hsa-miR-103b	Renamed hsa-miR-103b
hsa-miR-1201	hsa-miR-1201		Deleted in miRBase 16
hsa-miR-1259	hsa-miR-1259		Deleted in miRBase 16
	hsa-miR-1274a		Deleted in miRBase 17
	hsa-miR-1274b		Deleted in miRBase 17
	hsa-miR-1308		Deleted in miRBase 16
hsa-miR-1974			Deleted in miRBase 15
	hsa-miR-1975		Deleted in miRBase 15
	hsa-miR-1977		Deleted in miRBase 15
	hsa-miR-1978		Deleted in miRBase 15
hsa-miR-1979			Deleted in miRBase 16
	hsa-miR-2277	hsa-miR-2277-3	Renamed hsa-miR-2277-3
hsa-miR-220a	hsa-miR-220a		Deleted in miRBase 16
hsa-miR-220b	hsa-miR-220b		Deleted in miRBase 16
hsa-miR-220c	hsa-miR-220c		Deleted in miRBase 16
hsa-miR-453	hsa-miR-453	hsa-miR-323b-5p	Renamed hsa-miR-323b-5p
hsa-miR-500		hsa-miR-500a	Renamed hsa-miR-500a
hsa-miR-550	hsa-miR-550	hsa-miR-550a	Renamed hsa-miR-550a
hsa-miR-550-star		hsa-miR-550a_star	Renamed hsa-miR-550a_star
hsa-miR-642	hsa-miR-642	hsa-miR-642a	Renamed hsa-miR-642a
hsa-miR-675b		hsa-miR-675_star	Renamed hsa-miR-675_star
hsa-miR-886-3p	hsa-miR-886-3p		Deleted in miRBase 16
hsa-miR-886-5p	hsa-miR-886-5p		Deleted in miRBase 16
	hsa-miR-17 / hsa-miR-106a		Merged (Indistinguishable)
	hsa-miR-181b / hsa-miR-181d		Merged (Indistinguishable)

miRCURY	nCounter	SOLiD / Illumina	Comment
	hsa-miR-199a-3p / hsa-miR-199b-3p		Merged (Indistinguishable)
	hsa-miR-20a / hsa-miR-20b		Merged (Indistinguishable)
	hsa-miR-500 / hsa-miR-501-5p		Merged (Indistinguishable)
	hsa-miR-517c / hsa-miR-519a		Merged (Indistinguishable)
	hsa-miR-518a-5p / hsa-miR-520d-5p / hsa-miR-527		Merged (Indistinguishable)
	hsa-miR-518d-5p / hsa-miR-520c-5p / hsa-miR-526a		Merged (Indistinguishable)
