# Supplementary material for: Performance Comparison of Digital microRNA Profiling Technologies Applied on Human Breast Cancer Cell Lines
Source: PLoS One. 2013 Oct 8;8(10):e75813. doi: 10.1371/journal.pone.0075813 (PMC3793004; doi:10.1371/journal.pone.0075813)
Supplement: Table S2 — Sensitivity and specificity calculation. A positive miRNA was defined as a miRNA that were detected by at least 3 platforms. In total 777 miRNAs were defined as true positive and 1291 miRNAs were defined as true negatives. Only miRNAs screened for in all platforms were included. (A) nCounter, (B), miRCURY, (C) SOLiD, (D) Illumina, (E) Sensitivity and Specificity calculation. (PDF) [file pone.0075813.s005.pdf]

Supplementary Table S2: Sensitivity and specificity calculation. A positive miRNA was defined as a miRNA that were detected by at least 3 platforms. In total 777 miRNAs were defined as true positive and 1291 miRNAs were defined as true negatives. Only miRNAs screened for in all platforms were included. (A) nCounter, (B), miRCURY, (C) SOLiD, (D) Illumina, (E) Sensitivity and Specificity calculation.

(A) nCounter	Condition Positive	Condition Negative
Test positive	501	133
Test negative	276	1158

(B) miRCURY	Condition Positive	Condition Negative
Test positive	745	168
Test negative	32	1123

(C) SOLiD	Condition Positive	Condition Negative
Test positive	763	309
Test negative	14	982

(D) Illumina	Condition Positive	Condition Negative
Test positive	764	196
Test negative	13	1095

(E)	nCounter	miRCURY	SOLiD	Illumina
Sensitivity	0.645	0.959	0.982	0.983
Specificity	0.897	0.870	0.761	0.848
