# Supplementary material for: Performance Comparison of Digital microRNA Profiling Technologies Applied on Human Breast Cancer Cell Lines
Source: PLoS One. 2013 Oct 8;8(10):e75813. doi: 10.1371/journal.pone.0075813 (PMC3793004; doi:10.1371/journal.pone.0075813)
Supplement: Table S3 — IsomiR quantitated in NGS data. NGS data were mapped against Exma-miRDB (includes only the mature miRNA sequences in miRBase v17), as well as the hairpin sequences of all annotated miRNA in miRBase v17. Both sets of NGS data generated from Illumina and SOLiD were used for isomiR quantification. (PDF) [file pone.0075813.s006.pdf]

Supplementary Table S3: IsomiR quantitated in NGS data. NGS data were mapped against Exma-miRDB (includes only the mature miRNA sequences in miRBase v17), as well as the hairpin sequences of all annotated miRNA in miRBase v17. Both sets of NGS data generated from Illumina and SOLiD were used for isomiR quantification.

**SOLiD**	**Full miRBase v17 reference**	**Mature only reference**	**Present isomiRs**
SK-BR-3	14,056,154	6,125,461	56 %
AU565	9,989,478	4,805,757	52 %
Hs 578T	8,904,949	4,061,560	54 %
Hs 578Bst	17,828,685	9,088,681	49 %

**Illumina**	**Full miRBase v17 reference**	**Mature only reference**	**Present isomiRs**
SK-BR-3	18,629,691	8,235,782	56 %
AU565	12,986,479	7,275,786	44 %
Hs 578T	8,832,593	4,526,937	49 %
Hs 578Bst	16,243,203	8,846,983	46 %
