# Supplementary material for: Performance Comparison of Digital microRNA Profiling Technologies Applied on Human Breast Cancer Cell Lines
Source: PLoS One. 2013 Oct 8;8(10):e75813. doi: 10.1371/journal.pone.0075813 (PMC3793004; doi:10.1371/journal.pone.0075813)
Supplement: Table S4 — MicroRNA profile of two biological replicates using SOLiD sequencing. Values are log2 transformed and the differences are presented as absolute fold change values. (PDF) [file pone.0075813.s007.pdf]

Supplementary Table S4: MicroRNA profile of two biological replicates using SOLiD sequencing. Values are log_2 transformed and the differences are presented as absolute fold change values.

Name	Log2 Hs578T #1	Log2 Hs578T #2	Abs fold change
hsa-let-7a	14,192	14,812	1,5
hsa-let-7a_star	5,955	7,078	2,2
hsa-let-7a-2_star	8,172	8,158	1,0
hsa-let-7b	10,130	14,374	18,9
hsa-let-7c	4,196	6,586	5,2
hsa-let-7d	11,789	10,965	1,8
hsa-let-7d_star	1,328	7,793	88,4
hsa-let-7e	11,219	10,453	1,7
hsa-let-7e_star	2,591	6,347	13,5
hsa-let-7f	14,049	13,924	1,1
hsa-let-7g	14,176	13,700	1,4
hsa-let-7g_star	-0,994	-0,989	1,0
hsa-let-7i	7,388	8,654	2,4
hsa-let-7i_star	8,473	9,648	2,3
hsa-miR-1	0,814	2,297	2,8
hsa-miR-100	11,594	15,700	17,2
hsa-miR-100_star	8,355	6,226	4,4
hsa-miR-101	1,913	2,855	1,9
hsa-miR-103a	17,200	16,617	1,5
hsa-miR-103a-2_star	5,943	5,945	1,0
hsa-miR-106b	10,024	8,725	2,5
hsa-miR-106b_star	5,895	5,287	1,5
hsa-miR-107	12,056	11,842	1,2
hsa-miR-10a	6,216	8,048	3,6
hsa-miR-10b	2,864	5,347	5,6
hsa-miR-1180	0,328	4,310	15,8
hsa-miR-1197	5,833	3,917	3,8
hsa-miR-1226	-1,994	4,480	88,9
hsa-miR-1247	-1,994	0,722	6,6
hsa-miR-1249	4,636	7,079	5,4
hsa-miR-125a-3p	1,006	5,761	27,0
hsa-miR-125a-5p	12,363	13,582	2,3
hsa-miR-125b	16,251	16,673	1,3
hsa-miR-125b-1_star	4,545	7,470	7,6
hsa-miR-126	8,736	7,339	2,6
hsa-miR-126_star	7,534	8,388	1,8
hsa-miR-1267	6,249	5,760	1,4
hsa-miR-127-3p	2,530	7,237	26,1
hsa-miR-1270	2,707	6,085	10,4
hsa-miR-1271	6,661	5,412	2,4
hsa-miR-1273c	1,176	3,434	4,8

Name	Log2 Hs578T #1	Log2 Hs578T #2	Abs fold change
hsa-miR-128	9,225	9,545	1,2
hsa-miR-1280	-0,409	0,654	2,1
hsa-miR-1285	1,814	4,290	5,6
hsa-miR-1287	1,814	3,773	3,9
hsa-miR-129-3p	9,097	8,349	1,7
hsa-miR-129-5p	2,707	3,689	2,0
hsa-miR-1296	1,913	1,844	1,0
hsa-miR-1305	2,650	3,158	1,4
hsa-miR-130a	10,283	10,980	1,6
hsa-miR-130b	10,621	10,759	1,1
hsa-miR-130b_star	6,333	8,307	3,9
hsa-miR-132	5,245	5,846	1,5
hsa-miR-132_star	9,123	6,969	4,5
hsa-miR-1323	2,960	6,280	10,0
hsa-miR-134	5,449	10,370	30,3
hsa-miR-1343	0,006	3,564	11,8
hsa-miR-135b	5,390	5,050	1,3
hsa-miR-136	2,864	1,781	2,1
hsa-miR-136_star	8,206	6,285	3,8
hsa-miR-137	8,245	6,755	2,8
hsa-miR-138	11,151	11,982	1,8
hsa-miR-138-1_star	-0,994	3,249	18,9
hsa-miR-139-5p	3,216	5,226	4,0
hsa-miR-140-3p	3,707	6,808	8,6
hsa-miR-140-5p	7,468	8,164	1,6
hsa-miR-141	0,006	0,443	1,4
hsa-miR-143	13,082	12,679	1,3
hsa-miR-145	15,390	17,062	3,2
hsa-miR-145_star	9,101	7,334	3,4
hsa-miR-146a	6,647	10,545	14,9
hsa-miR-148a	3,216	5,502	4,9
hsa-miR-148b	7,784	7,321	1,4
hsa-miR-149	8,439	10,178	3,3
hsa-miR-150	-1,994	3,814	56,0
hsa-miR-151-3p	6,727	9,437	6,5
hsa-miR-151-5p	5,433	12,760	160,6
hsa-miR-152	8,719	9,878	2,2
hsa-miR-153	2,864	2,376	1,4
hsa-miR-154	7,130	6,582	1,5
hsa-miR-154_star	8,939	8,320	1,5
hsa-miR-155	6,883	8,455	3,0
hsa-miR-155_star	5,781	6,537	1,7
hsa-miR-15a	4,984	6,123	2,2
hsa-miR-15a_star	8,921	4,830	17,0

Name	Log2 Hs578T #1	Log2 Hs578T #2	Abs fold change
hsa-miR-15b	14,477	14,694	1,2
hsa-miR-15b_star	9,703	8,595	2,2
hsa-miR-16	12,283	11,110	2,3
hsa-miR-16-2_star	4,514	1,196	10,0
hsa-miR-17	13,259	11,224	4,1
hsa-miR-17_star	7,310	4,967	5,1
hsa-miR-181a	12,199	12,298	1,1
hsa-miR-181a_star	12,228	9,389	7,2
hsa-miR-181a-2_star	7,933	9,184	2,4
hsa-miR-181b	6,696	9,173	5,6
hsa-miR-181c	6,632	5,399	2,4
hsa-miR-181d	3,761	3,995	1,2
hsa-miR-182	11,152	10,916	1,2
hsa-miR-183	8,512	8,590	1,1
hsa-miR-185	13,017	12,429	1,5
hsa-miR-186	7,636	9,968	5,0
hsa-miR-186_star	3,364	3,203	1,1
hsa-miR-188-5p	5,156	6,667	2,8
hsa-miR-18a	7,449	7,207	1,2
hsa-miR-18a_star	2,960	4,104	2,2
hsa-miR-190	1,466	3,566	4,3
hsa-miR-191	9,893	11,043	2,2
hsa-miR-191_star	3,254	6,547	9,8
hsa-miR-192	10,989	11,647	1,6
hsa-miR-192_star	2,530	2,095	1,4
hsa-miR-193a-3p	10,361	8,981	2,6
hsa-miR-193a-5p	8,117	11,666	11,7
hsa-miR-193b	10,754	10,766	1,0
hsa-miR-193b_star	4,416	8,501	17,0
hsa-miR-194	2,254	4,478	4,7
hsa-miR-195	3,364	5,142	3,4
hsa-miR-195_star	4,901	3,865	2,1
hsa-miR-196a	6,235	11,283	33,1
hsa-miR-196b	3,889	8,394	22,7
hsa-miR-197	7,184	12,796	48,9
hsa-miR-199a-3p//hsa-miR-199b-3p	14,146	13,044	2,1
hsa-miR-199a-5p	15,210	12,943	4,8
hsa-miR-199b-5p	6,700	3,775	7,6
hsa-miR-19a	12,344	9,827	5,7
hsa-miR-19b	13,063	11,409	3,1
hsa-miR-19b-1_star	7,169	5,118	4,1
hsa-miR-200a	3,734	2,781	1,9
hsa-miR-200c	-1,994	1,297	9,8
hsa-miR-203	3,984	0,909	8,4

Name	Log2 Hs578T #1	Log2 Hs578T #2	Abs fold change
hsa-miR-204	10,656	9,221	2,7
hsa-miR-20a	12,837	9,570	9,6
hsa-miR-21	15,694	14,578	2,2
hsa-miR-21_star	6,751	8,082	2,5
hsa-miR-210	5,310	8,020	6,5
hsa-miR-212	1,814	1,116	1,6
hsa-miR-214_star	5,301	2,837	5,5
hsa-miR-218	10,777	9,172	3,0
hsa-miR-219-1-3p	0,006	1,490	2,8
hsa-miR-22	11,400	11,765	1,3
hsa-miR-22_star	10,317	9,501	1,8
hsa-miR-221	9,128	12,814	12,9
hsa-miR-221_star	6,846	4,967	3,7
hsa-miR-222	9,927	10,951	2,0
hsa-miR-223	2,864	5,802	7,7
hsa-miR-224	4,889	5,655	1,7
hsa-miR-2278	0,006	2,376	5,2
hsa-miR-23a	14,042	13,570	1,4
hsa-miR-23a_star	4,433	4,537	1,1
hsa-miR-23b	12,190	11,814	1,3
hsa-miR-23b_star	1,176	1,761	1,5
hsa-miR-24	14,912	15,325	1,3
hsa-miR-24-1_star	1,913	4,422	5,7
hsa-miR-24-2_star	1,006	2,603	3,0
hsa-miR-25	10,039	12,180	4,4
hsa-miR-26a	9,732	13,277	11,7
hsa-miR-26a-1_star	6,146	6,876	1,7
hsa-miR-26a-2_star	3,889	5,412	2,9
hsa-miR-26b	5,978	8,075	4,3
hsa-miR-26b_star	6,110	5,706	1,3
hsa-miR-27a	14,705	12,581	4,4
hsa-miR-27a_star	8,469	10,084	3,1
hsa-miR-27b	10,427	9,685	1,7
hsa-miR-27b_star	5,474	7,702	4,7
hsa-miR-28-3p	4,364	10,557	73,2
hsa-miR-28-5p	4,679	4,939	1,2
hsa-miR-296-3p	2,707	2,201	1,4
hsa-miR-296-5p	7,096	8,460	2,6
hsa-miR-299-3p	7,102	7,108	1,0
hsa-miR-299-5p	8,822	8,933	1,1
hsa-miR-29a	16,506	14,090	5,3
hsa-miR-29a_star	4,399	4,516	1,1
hsa-miR-29b	16,983	15,634	2,5
hsa-miR-29b-1_star	6,445	9,531	8,5

Name	Log2 Hs578T #1	Log2 Hs578T #2	Abs fold change
hsa-miR-29c	9,768	8,797	2,0
hsa-miR-29c_star	1,176	-0,478	3,1
hsa-miR-301a	10,038	7,144	7,4
hsa-miR-301b	7,240	5,441	3,5
hsa-miR-30a	8,583	7,919	1,6
hsa-miR-30a_star	6,381	7,144	1,7
hsa-miR-30b	13,885	11,413	5,5
hsa-miR-30c	11,118	10,420	1,6
hsa-miR-30c-2_star	2,761	2,893	1,1
hsa-miR-30d	7,551	8,728	2,3
hsa-miR-30d_star	3,814	5,167	2,6
hsa-miR-30e	7,204	6,455	1,7
hsa-miR-30e_star	3,136	0,849	4,9
hsa-miR-31	14,756	15,814	2,1
hsa-miR-31_star	7,238	5,849	2,6
hsa-miR-3129-3p	1,176	4,282	8,6
hsa-miR-3145-5p	3,839	2,967	1,8
hsa-miR-3152-5p	2,530	2,704	1,1
hsa-miR-32	5,530	4,468	2,1
hsa-miR-320a	9,184	11,949	6,8
hsa-miR-320b	-0,994	3,123	17,3
hsa-miR-320d	0,328	4,408	16,9
hsa-miR-323-3p	6,319	9,455	8,8
hsa-miR-323b-3p	3,364	1,341	4,1
hsa-miR-324-5p	7,934	9,113	2,3
hsa-miR-326	2,707	3,380	1,6
hsa-miR-328	5,538	9,010	11,1
hsa-miR-329	8,893	8,370	1,4
hsa-miR-330-3p	4,561	7,949	10,5
hsa-miR-331-3p	8,450	8,819	1,3
hsa-miR-335	4,801	6,242	2,7
hsa-miR-335_star	2,913	4,998	4,2
hsa-miR-337-3p	10,338	9,927	1,3
hsa-miR-337-5p	9,646	7,743	3,7
hsa-miR-339-3p	4,235	3,702	1,4
hsa-miR-339-5p	9,658	9,290	1,3
hsa-miR-33a	7,597	4,936	6,3
hsa-miR-33b	5,416	3,058	5,1
hsa-miR-340	5,506	5,698	1,1
hsa-miR-340_star	5,966	5,462	1,4
hsa-miR-342-3p	10,200	9,423	1,7
hsa-miR-345	2,913	8,706	55,4
hsa-miR-34a	9,689	8,255	2,7
hsa-miR-34c-5p	9,536	7,029	5,7

Name	Log2 Hs578T #1	Log2 Hs578T #2	Abs fold change
hsa-miR-3605-3p	2,650	4,593	3,8
hsa-miR-3605-5p	1,913	2,643	1,7
hsa-miR-361-3p	5,628	4,503	2,2
hsa-miR-361-5p	6,981	8,498	2,9
hsa-miR-3613-5p	5,614	1,879	13,3
hsa-miR-362-3p	8,013	5,562	5,5
hsa-miR-362-5p	6,883	5,758	2,2
hsa-miR-365	8,806	10,102	2,5
hsa-miR-3676	7,072	7,304	1,2
hsa-miR-369-3p	6,952	8,905	3,9
hsa-miR-369-5p	7,828	6,515	2,5
hsa-miR-370	5,989	11,653	50,7
hsa-miR-373	3,094	2,239	1,8
hsa-miR-374a	7,707	8,117	1,3
hsa-miR-374a_star	5,858	5,524	1,3
hsa-miR-374b	7,458	10,238	6,9
hsa-miR-374b_star	5,814	4,824	2,0
hsa-miR-376a	9,997	12,012	4,0
hsa-miR-376a_star	4,801	5,064	1,2
hsa-miR-376b	3,621	7,271	12,6
hsa-miR-376c	12,157	12,645	1,4
hsa-miR-377	2,399	4,396	4,0
hsa-miR-377_star	4,364	4,119	1,2
hsa-miR-378	8,337	8,491	1,1
hsa-miR-378_star	8,261	7,281	2,0
hsa-miR-379	9,147	10,658	2,8
hsa-miR-379_star	4,196	5,567	2,6
hsa-miR-381	9,049	6,714	5,0
hsa-miR-382	9,010	10,384	2,6
hsa-miR-3909	2,094	2,358	1,2
hsa-miR-3940-3p	2,094	6,544	21,9
hsa-miR-409-3p	11,504	12,557	2,1
hsa-miR-409-5p	5,561	5,406	1,1
hsa-miR-410	7,807	7,498	1,2
hsa-miR-411	-0,994	-0,989	1,0
hsa-miR-411_star	3,498	3,576	1,1
hsa-miR-421	6,273	9,664	10,5
hsa-miR-423-3p	6,191	9,637	10,9
hsa-miR-423-5p	-1,994	5,951	246,4
hsa-miR-424	5,176	3,990	2,3
hsa-miR-425	12,325	10,337	4,0
hsa-miR-425_star	7,211	7,384	1,1
hsa-miR-4286	10,133	10,698	1,5
hsa-miR-431	8,401	8,477	1,1

Name	Log2 Hs578T #1	Log2 Hs578T #2	Abs fold change
hsa-miR-433	4,254	5,666	2,7
hsa-miR-449a	2,399	4,298	3,7
hsa-miR-451	2,591	9,279	103,1
hsa-miR-452	6,268	3,857	5,3
hsa-miR-454_star	7,781	8,370	1,5
hsa-miR-455-3p	6,934	7,725	1,7
hsa-miR-455-5p	5,506	4,314	2,3
hsa-miR-4636	3,433	3,592	1,1
hsa-miR-4676-5p	0,328	1,289	1,9
hsa-miR-4677-3p	5,125	4,908	1,2
hsa-miR-4731-3p	3,051	3,325	1,2
hsa-miR-4746-5p	3,889	3,869	1,0
hsa-miR-484	11,497	13,368	3,7
hsa-miR-485-3p	7,348	8,190	1,8
hsa-miR-486-3p	-0,994	6,697	206,6
hsa-miR-486-5p	-1,994	6,894	473,7
hsa-miR-487a	8,235	7,157	2,1
hsa-miR-487b	9,820	10,040	1,2
hsa-miR-490-5p	-1,994	2,454	21,8
hsa-miR-491-5p	1,006	3,992	7,9
hsa-miR-493_star	9,877	6,629	9,5
hsa-miR-494	8,012	6,908	2,2
hsa-miR-495	11,762	9,909	3,6
hsa-miR-496	4,721	4,615	1,1
hsa-miR-497	6,506	6,363	1,1
hsa-miR-497_star	2,254	4,081	3,5
hsa-miR-498	2,176	4,545	5,2
hsa-miR-500a	4,814	1,938	7,3
hsa-miR-500a_star	-1,994	4,929	121,4
hsa-miR-502-3p	-0,994	6,873	233,3
hsa-miR-503	-0,994	1,855	7,2
hsa-miR-505	8,176	7,202	2,0
hsa-miR-505_star	3,292	4,649	2,6
hsa-miR-512-3p	5,482	6,214	1,7
hsa-miR-515-3p	4,913	6,397	2,8
hsa-miR-515-5p	1,913	2,989	2,1
hsa-miR-516b	3,591	3,545	1,0
hsa-miR-517_star	4,864	5,088	1,2
hsa-miR-517a	9,192	9,403	1,2
hsa-miR-517b	2,176	2,852	1,6
hsa-miR-517c	6,748	6,318	1,3
hsa-miR-518b	3,621	9,010	41,9
hsa-miR-518e	4,006	3,978	1,0
hsa-miR-518f_star	3,051	3,781	1,7

Name	Log2 Hs578T #1	Log2 Hs578T #2	Abs fold change
hsa-miR-519a	3,364	5,407	4,1
hsa-miR-519b-3p	0,006	6,518	91,2
hsa-miR-519d	1,707	3,012	2,5
hsa-miR-520c-3p	0,591	8,186	193,3
hsa-miR-520g	5,416	5,489	1,1
hsa-miR-520h	5,346	6,100	1,7
hsa-miR-522	3,839	4,880	2,1
hsa-miR-524-3p	-0,994	2,499	11,3
hsa-miR-524-5p	3,216	4,772	2,9
hsa-miR-525-3p	0,591	3,883	9,8
hsa-miR-525-5p	1,591	2,891	2,5
hsa-miR-526b	5,346	4,897	1,4
hsa-miR-532-3p	3,734	6,652	7,6
hsa-miR-532-5p	7,867	11,661	13,9
hsa-miR-539	4,937	7,248	5,0
hsa-miR-543	10,726	9,269	2,7
hsa-miR-545_star	6,381	4,680	3,3
hsa-miR-548d-3p	3,292	3,712	1,3
hsa-miR-548k	0,814	2,343	2,9
hsa-miR-548o	2,814	1,938	1,8
hsa-miR-549	1,328	2,430	2,1
hsa-miR-550a	2,913	5,458	5,8
hsa-miR-550a_star	6,721	5,487	2,4
hsa-miR-551a	3,707	3,407	1,2
hsa-miR-551b	4,949	5,742	1,7
hsa-miR-561	2,913	2,593	1,2
hsa-miR-570	6,462	4,764	3,2
hsa-miR-574-3p	13,353	15,194	3,6
hsa-miR-574-5p	4,877	8,825	15,4
hsa-miR-576-5p	2,707	2,781	1,1
hsa-miR-582-3p	-0,409	3,647	16,6
hsa-miR-582-5p	5,700	5,185	1,4
hsa-miR-589_star	0,814	3,132	5,0
hsa-miR-590-3p	8,476	10,280	3,5
hsa-miR-590-5p	12,927	9,976	7,7
hsa-miR-598	7,789	6,233	2,9
hsa-miR-615-3p	1,591	5,798	18,5
hsa-miR-615-5p	-0,409	-0,989	1,5
hsa-miR-616_star	4,925	2,722	4,6
hsa-miR-618	3,176	2,544	1,5
hsa-miR-624_star	3,433	2,664	1,7
hsa-miR-625	3,094	3,909	1,8
hsa-miR-625_star	9,440	9,542	1,1
hsa-miR-627	3,136	5,600	5,5

Name	Log2 Hs578T #1	Log2 Hs578T #2	Abs fold change
hsa-miR-628-5p	3,761	6,281	5,7
hsa-miR-629	5,245	5,122	1,1
hsa-miR-629_star	2,328	4,742	5,3
hsa-miR-641	6,784	4,627	4,5
hsa-miR-642a	0,591	3,238	6,3
hsa-miR-654-3p	4,216	5,677	2,8
hsa-miR-654-5p	2,006	2,972	2,0
hsa-miR-655	7,703	7,552	1,1
hsa-miR-656	6,738	7,835	2,1
hsa-miR-660	6,171	5,367	1,7
hsa-miR-664	4,889	4,499	1,3
hsa-miR-671-3p	5,072	7,548	5,6
hsa-miR-671-5p	1,814	-0,478	4,9
hsa-miR-7	8,112	7,408	1,6
hsa-miR-7-1_star	7,886	7,845	1,0
hsa-miR-708	1,328	7,226	59,6
hsa-miR-720	10,225	9,683	1,5
hsa-miR-744	4,094	9,438	40,6
hsa-miR-758	2,006	2,553	1,5
hsa-miR-766	1,707	3,672	3,9
hsa-miR-769-3p	3,176	4,207	2,0
hsa-miR-769-5p	6,931	5,932	2,0
hsa-miR-874	0,006	3,967	15,6
hsa-miR-877_star	2,399	3,913	2,9
hsa-miR-887	-0,994	2,513	11,4
hsa-miR-889	4,545	4,126	1,3
hsa-miR-92a	7,779	12,008	18,7
hsa-miR-92a-1_star	0,006	6,109	68,7
hsa-miR-92b	5,441	9,192	13,5
hsa-miR-93	6,561	9,729	9,0
hsa-miR-93_star	6,466	8,063	3,0
hsa-miR-935	3,650	8,163	22,8
hsa-miR-940	5,650	8,729	8,4
hsa-miR-944	-1,994	2,471	22,1
hsa-miR-96	10,191	10,278	1,1
hsa-miR-98	11,822	9,959	3,6
hsa-miR-99a	7,807	8,965	2,2
hsa-miR-99b	9,731	12,902	9,0
hsa-miR-99b_star	5,407	8,093	6,4
Median fold change			*2,6*
